# Metabolic syndrome increases the length of stay and medical complications after hip and knee arthroplasty: results from a prospective cohort study of 2,901 patients

**DOI:** 10.2340/17453674.2024.42112

**Published:** 2024-10-14

**Authors:** Rasmus Reinholdt SØRENSEN, Signe TIMM, Lasse Enkebølle RASMUSSEN, Claus Lohman BRASEN, Claus VARNUM

**Affiliations:** 1 Department of Orthopaedic Surgery, Lillebaelt Hospital, University Hospital of Southern Denmark; 2 Department of Regional Health Research, University of Southern Denmark; 3 Department of Immunology and Biochemistry, Lillebaelt Hospital, University Hospital of Southern Denmark, Denmark

## Abstract

**Background and purpose:**

Metabolic syndrome (MetS) affects more than 60% of the patients having a hip or knee arthroplasty due to osteoarthritis. As it is debated whether metabolic syndrome increases the risk of complications, we aimed to investigate the length of stay (LOS) and risk of readmission at 30 and 90 days after surgery, including causes of readmission.

**Methods:**

We conducted a prospective cohort study of 2,901 patients undergoing hip and knee arthroplasty from May 2017 to November 2019. Physical examination, blood samples, and medical history from national registries determined the diagnosis of metabolic syndrome from the International Diabetes Federation definition. We used multivariate linear regression to investigate differences in LOS according to MetS, and binary regression to investigate the risk and causes of readmission within 30 and 90 days, including 95% confidence intervals (CI) and P values.

**Results:**

Patients with MetS showed a slightly longer LOS (0.20 days, CI 0.10–0.29) and had an increased risk of readmission within 90 days (adjusted relative risk [RR] 1.2, CI 1.0–1.4; P = 0.02), but not within 30 days (adjusted RR 1.1, CI 0.9–1.4; P = 0.3) after surgery. Cardiovascular disease was the dominant cause of readmission.

**Conclusion:**

Although patients with MetS do not experience a clinically relevant longer LOS after hip and knee arthroplasty, they have an increased risk of 90-day readmission mainly due to cardiovascular complications, which should be considered when planning surgical care in this group of patients.

Fast-track surgery and optimized recovery in patients undergoing hip and knee arthroplasty has been implemented with great success in Denmark [1] and this protocol has been adopted by several countries around the world. Fast-track protocols have reduced the length of stay (LOS) without increasing the risk of complications and readmissions but there has been no focus on metabolic syndrome (MetS) [2]. The influence of obesity and diabetes has been investigated thoroughly in relation to complications after hip and knee arthroplasty [3,4]. MetS is a disease that comprises different risk factors highly related to lifestyle behavior (Table 1), influencing the risk of developing cardiovascular disease and diabetes mellitus type 2 [5] The prevalence of MetS is 5 times higher in patients with osteoarthritis (OA) [6], and MetS is assumed to be one of the largest challenges in modern healthcare [7]. Previous studies have identified MetS as a dominant risk factor for postoperative complications and readmissions after hip and knee arthroplasty [8], but a dominance of retrospective studies and a variation in the definitions used makes direct comparison difficult. As the focus on healthcare expenses has increased during recent years, in some countries with economic bonuses or penalties related to qualitative measurements [9,10] the urge to prevent expensive days in hospital and readmissions has been intensified. We have previously shown that MetS does not increase the mortality or the risk of revision surgery in patients undergoing hip and knee arthroplasty [11]. To our knowledge, investigation of the association between MetS and LOS, readmission, and cause of medical complications has never been conducted in a fast-track hip and knee arthroplasty population. We investigated the LOS comparing patients who have MetS with patients who do not have MetS. Furthermore, we studied the relative risk of readmission within 30 and 90 days, and causes thereof within the 2 groups.

**Table 1:** International Diabetes Foundation metabolic syndrome definition modified with ethnic specific values of abdominal circumference for a European population and taking into account prior diagnoses and redeemed medication

Factor	Measurements, diagnoses or redeemed medication
Central obesity, defined as	Abdominal circumference > 94 cm for men and > 80 cm for women, or body mass index > 30 for both sexes
*Plus any 2 of 4:*	
Elevated fasting triglycerides	≥ 1.7 mmol/L
Decreased fasting level of high-density lipoprotein	
	Men < 1.03 mmol/L
	Women < 1.29 mmol/L
Hypertension	Systolic blood pressure ≥ 130 mmHg
	or diastolic blood pressure ≥ 85 mmHg
	Diagnoses: ICD-10 codes I10–15
	Medication: ATC codes C02A-C, C02DA, C02L, C02L, C03A-B, C03D-E, C03X, C07, C08 or C09
Elevated fasting P-glucose	
	≥ 5.6 mmol/L
	Diagnoses: ICD-10 code E11
	Medication: ATC-codes A10A or A10B

## Methods

In Denmark, all 5.9 million residents benefit from free tax-funded healthcare from general practitioners and hospitals and a unique 10-digit personal identification number allows unambiguous linkage between Danish healthcare registries.

### Study population

**Figure 1:**
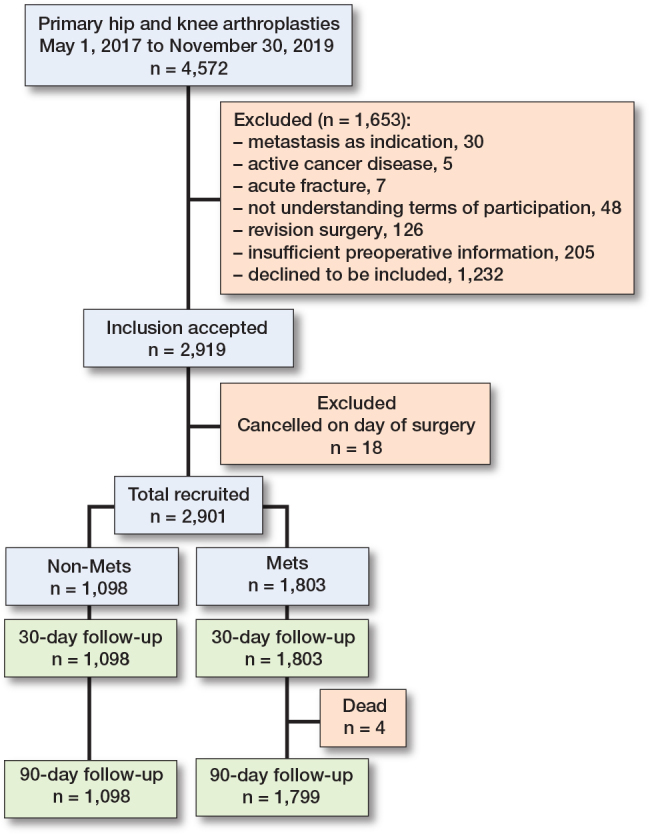
Flowchart of the study population.

All patients undergoing primary total hip or knee arthroplasty (THA or TKA), as well as unicompartmental knee arthroplasty (UKA) at our institution in the period of May 1, 2017 to November 30, 2019 were screened for inclusion. We excluded patients who had advanced or end-stage cancer and patients receiving an arthroplasty because of cancer or metastasis in the bony structures around the joint, as well as patients having acute fractures of the affected limb and patients not willing or able to give informed consent (Figure 1). The surgical procedures are standardized within the department by a fast-track protocol. This involves multimodal opioid-sparing analgesia, early postoperative mobilization (within 6 hours) assisted by the physiotherapist and nursing staff, and discharge to own home based on functional criteria [12]. All patients are intravenously administered 125 mg methylprednisolone preoperatively and 1 g of tranexamic acid intraoperatively. Prior to surgery, obese patients were advised to lose weight and perform exercise, but these were not criteria for surgery. THA was performed through a posterior approach, while TKA and UKA were performed from a medial parapatellar approach. A tourniquet was applied in all UKAs, while it was applied according to the surgeons’ preference in TKA. In UKA and TKA, intraoperative high-volume local infiltration analgesia (LIA) was administered.

### Data sources

Data was obtained from several registries unique to the Danish healthcare system. The Danish National Registry of Patients (DNRP) contains data on all discharges from hospitals in Denmark, including dates of admission and discharge, surgical procedures performed and up to 20 diagnoses for every discharge, assigned by the physician discharging the patient [13]. The DNRP was used to identify diagnoses related to MetS, collecting comorbidity data on all patients to construct the Charlson Comorbidity Index [14] and investigating readmission and related diagnoses at 30 and 90 days’ follow-up. Combined with our local database on patients having hip and knee arthroplasty, data on LOS was obtained.

The Register of Pharmaceutical Sales (RPS) was used to identify the use of drugs related to MetS. The RPS contains an electronic record with information related to the user, the prescriber, the pharmacy, and the dispensed drug, each time a prescription is redeemed at a pharmacy since its establishment in 2004 [15].

The Danish Hip Arthroplasty Registry (DHR) and the Danish Knee Arthroplasty Registry (DKR) were established with the aim of registering and improving the results after hip and knee arthroplasty in Denmark. The DHR and DKR contain information on nationwide primary operations, revisions, and postoperative complications, and are validated and valuable tools for quality improvement and research [16-18].

### Metabolic syndrome

MetS was defined using the International Diabetes Foundation (IDF) consensus [19]. A physical examination was performed at inclusion, measuring weight, height, waist circumference, and blood pressure. Blood samples were drawn to analyze plasma fasting triglyceride, high-density lipoprotein (HDL), total cholesterol, and fasting glucose on the day of surgery as pre-surgery overnight fasting samples. Total cholesterol, HDL, and triglycerides were sampled using lithium heparin anticoagulated vacutainer tubes, centrifuged for 10 minutes at 2,000 g and analyzed on a Cobas 8000 c702 (Roche, Basel, Switzerland) in an ISO 15189 accredited hospital laboratory. As a modification to the IDF definition, we included prior diagnoses and medicine consumption in a 10-year period leading up to the date of surgery (Table 1). This approach was applied in an attempt to minimize the risk of recall bias introduced by the patient when self-reporting their health condition.

### Medical complications

Several diagnoses can be assigned on admission in the DNRP. One diagnosis is considered the main cause of readmission, and from that the readmissions were categorized into 7 types of events, according to the International Classification of Diseases and Related Health Problems, 10th edition (ICD-10) (Table 2, see Appendix). Thromboembolism was composed of lung embolism and venous thromboembolism. Stroke represented stroke only, whereas cardiovascular complication was defined as acute myocardial infarction, heart failure, hypertension, cardiac arrhythmias, heart valve disease, cardiac inflammation, and infection and aorta disease. Pulmonary complication was defined as pneumonia and other respiratory tract infections. Renal complication was defined as any renal disease. Urinary tract infection was defined as an event with the diagnosis of urinary tract infection. Other infectious complications were defined as any infection involving bacterial or viral genesis including bacteremia and sepsis, but without pneumonia, respiratory tract infection, or urinary tract infection. Complications were recorded only if the patient needed hospitalization for at least 12 hours.

**Table 2:** Classification of disease categories used in the analysis of readmission

Category/Type of disease	ICD-10 codes
Thromboembolism	
Lung embolism	I26
Venous embolism	I80.1-3
Stroke	I63–I64, G45.9
Cardiovascular	
Acute myocardial infarction and angina pectoris	I20.0, I20, I21, I25.1, I25.9
Heart failure	I50.0–I50.3, I50.8, I50.9, I11.0, I13.0, I13.2, I42.0, I42.6–I42.9
Hypertension	I10–I15
Cardiac arrhythmias	I44.0, I44.1, I44.2, I44.3, I45.5A, I45.5B, I45.5C, I45.5G, I47.0, I47.2, I48, I49.0
Heart valve disease	I05, I06, I34, I35, I39.0, I39.1, I51.1A
Cardiac inflammation and infection	I00–I02, I30–I33, I38, I39.8, I40, I41, I51.4, I09.0, B37.6
Aorta disease	I71.0–I71.6, I71.8–I71.9
Pulmonary disease	
Upper respiratory tract infection	J00–J06, J36, J39.0, J39.1
Pneumonia	J12–J18
Other lower respiratory tract infection	J20–J22, J44.0, J85.1, J86, J34.0, J35.0, J38.3C, J38.3D, J38.7B, J38.7F, J38.7G
Renal disease	N00–N05, N07, N08, N11, N14–N16, N18–N19, N26, N27, E10.2, E11.2, Q61.1–Q61.4
Urinary tract infections	
Urinary tract infection	N10, N12, N30, N33.0, N34, N39.0, N13.6, N28.8D, N28.8E, N28.8F, N29.0, N29.1
Other infections	
Miscellaneous bacterial infection	A20–A38, A42–A44, A48–A49, A65–A79
Tuberculosis	A15–A19
Atypical mycobacteria	A31
Bacteremia	A49.9, A39.4
Sepsis	A40–A41, A37.7, A54.8G, A02.1, A22.7, A26.7, A42.7, A28.2B, B37.7,
Abscess	A06.5, A54.1, B43, D73.3, E06.0A, E23.6A, E32.1, G06, G07, H00.0A, H05.0A, H44.0A, H60.0, J34.0A, J36, J38.3, J38.7G, J39.0, J39.1, J39.8A, J85.1, J85.2, J85.3, K04.6,K04.7, K11.3, K12.2, K13.0A, K14.0A, K20.9A, K35.3A, K35.3B, K57.0, K57.2, K57.4, K57.8, K61, K63.0, K65.0, K75.0, K81.0A, K85.8A, L02, L05.0, L05.9, M60.8A, M86.8A, M86.9A, N15.1, N34.0, N41.2, N45.0, N48.2, N49.2A, N61.9A, N61.9B, N70.0A, N70.0B, N71.0A, N73.0A, N73.0B, N73.2A, N73.2B, N73.3A, N73.5A, N73.8A, N73.8C, N75.1, N76.4, N76.8A
Skin infection	A46, H01.0, H03, H60.0, H60.1, H60.2, H60.3, H62, K12.2, K13.0, K61, M72.6, L01, L08
Meningitis	G00, G01, G02, G03, A32.1, A39.0, A17.0, A20.3, A87, A54.8D, A02.2C, B37.5, B00.3, B01.0, B02.1, B05.1, B26.1, B38.4
Intra-abdominal and gastrointestinal infection	A00–A09, K35, K37, K57.0, K57.2, K57.4, K57.8, K61, K63.0, K65.0, K65.9, K67, K75.0, K75.1, K80.0, K80.3, K80.4, K81.0, K81.9, K83.0, K85.9
Male genital infection	N41, N45, N48.1, N48.2, N49, N51.1, N51.2
Obstetrical and female pelvic infection	N70–N77, O23, O26.4, O41.1, O74.0, O75.3, O85, O86, O88.3, O91, O98
Infectious complications of procedures	T80.2, T81.4, T82.6, T82.7, T83.5, T83.6, T84.5, T84.6, T84.7, T85.7, T88.0, T89.9
Other infections or sequelae	B90–B99, K04.0, K05.2
Miscellaneous viral infection	A90–A99, B03–B09, B25–B34
HIV	B20–B24
Viral hepatitis	B15–B19
Influenza	J10–J11
Viral CNS infection	G00–G07, A80–A89

### Statistics

Descriptive statistics were used for demographic characteristics reporting the marginal mean and standard deviation (SD) for continuous measures or absolute numbers and proportions (%) for categorical measures. Patients without MetS were used as controls. The 2 groups were compared using standardized differences and values > 0.2 were considered an indication of imbalance [20].

**Figure 2:**
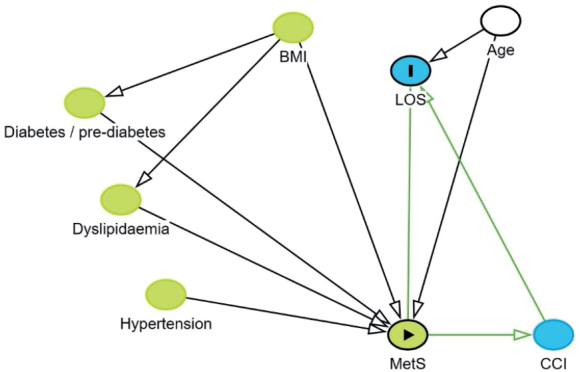
Directed acyclic graphs were used to determine the minimal adjustment set.

The primary outcome was LOS, and secondary outcomes were readmission within 30 and 90 days. Directed acyclic graphs (Figure 2, see Appendix) were used to determine the minimal adjustment set. The DAG included the following covariates: hypertension, diabetes/prediabetes, dyslipidemia, central obesity (defined in Table 1), body mass index (BMI), metabolic syndrome, age, and Charlson Comorbidity Index (CCI), and the minimal adjustment set included only age. All analyses were stratified by joint (hip/knee). Multivariate linear regression was used to estimate the predicted difference in LOS between groups with corresponding 95% confidence interval (CI) and P value [21]. Residuals were plotted in a histogram to verify the assumption of normal distribution, which was accepted. Furthermore, residuals vs fitted values were visualized in a scatter plot. Binary regression was used to estimate the relative risk (RR) of readmission within 30 and 90 days with corresponding 95% CI and P value. Analyses were performed as complete case, as missing data was 0.14%. Although patients lost to follow-up may not be missing completely at random, we consider potential bias from this to be with minimal impact on the results. Patients having more than one surgery on independent occasions or who had simultaneous bilateral arthroplasty were statistically handled by using robust variance modeling in order to account for non-independency in observations. All statistical analyses were performed using STATA version 18 (StataCorp LLC, College Station, TX, USA).

This paper follows the guidelines of Strengthening the Reporting of Observational Studies in Epidemiology (STROBE).

### Ethics, funding, and disclosures

All patients provided signed informed consent prior to inclusion. The study was conducted in accordance with the Declaration of Helsinki and approved by the Regional Committees on Health Research Ethics of Southern Denmark (record number S-20150105) and the Danish Data Protection Agency (record number 2008-58-0035). Funding was received from Region of Southern Denmark and the Johan Boserup and Lise Boserup scholarship. The financial support had no influence on the study design, data collection, or interpretation of data. CV and LER received travel expenses from Stryker with no relevance to the present study. LER received an institutional grant for research purposes from Stryker with no relevance to the present study. The authors have no conflict of interest related to this study. Complete disclosure of interest forms according to ICMJE are available on the article page, doi: 10.2340/17453674.2024.42112

## Results

2,901 independent procedures were included (Figure 1). Patients could be included more than once if they met the inclusion criteria with separate joints on independent occasions. 123 patients had simultaneous bilateral surgeries performed. MetS was present in 62% of the cohort (Table 3), which included more women and the indication for surgery was dominated by OA. The MetS group presented with a higher degree of comorbidity and overweight, as would be expected, while the age was similar between the 2 groups (Tables 3 and 4, see Appendix). The mean LOS among patients with MetS was 1.7 days (SD 1.6) and 1.5 days (SD 1.1) among patients without MetS. Between groups, there was a difference in LOS of 0.20 days (CI 0.10–0.29; P < 0.001) (Table 5), mainly caused by knee patients (0.16 days, CI 0.01–0.31), P = 0.04) when stratifying by joint (Table 6, see Appendix). While the mean and median LOS were similar, the range in which patients stayed in hospital after surgery had a wide span of up to 28 days in the MetS group, compared with a maximum of 9 days in the non-MetS group. Investigating the possibility of this being outliers, analysis showed that 73 MetS patients had a LOS of 5 or more days, compared with 25 patients in the non-MetS group.

**Table 3:** Demographics of the study population. Values are count [%] unless otherwise specified

Factor	MetS	Non-MetS	Standardized difference
Cases	1,803 [62]	1,098 [38]	
Age ^a^	68.0 (9.3)	67.6 (10.3)	–0.04
Female sex	1,012 [56]	604 [55]	0.02
Indication			
Primary osteoarthritis	1,569 [87]	915 [83]	–0.1
Other	220 [12]	177 [16]	0.1
Missing	14 [0.8]	6 [0.5]	–0.03
Charlson Index			
Low, 0	1,293 [72]	810 [74]	0.05
Medium, 1–2	394 [22]	242 [22]	0.0
High, ≥ 3	116 [6.4]	46 [4.2]	–0.1
Body mass index ^a^	30.4 (5.1)	25.9 (3.9)	–1.0
Missing	11 [0.6]	5 [0.5]	–0.02
Abdominal circumference, cm ^a^	104.3 (11.9)	91.7 (11.7)	–1.1
Missing	24 [1.3]	45 [4.2]	0.2
Factors of metabolic syndrome			
Elevated fasting triglycerides	642 [36]	63 [5.7]	–0.8
Decreased high-density lipoprotein	398 [22]	38 [3.5]	–0.6
Hypertension	1,759 [98]	850 [77]	–0.6
Elevated fasting P-glucose or diabetes	1,669 [93]	380 [35]	–1.5

a Mean (SD).

**Table 4:** Demographics of the study population, stratified by joint. Values are count [%] unless otherwise specified

	Hip procedures (n = 1,369)			Knee procedures (n = 1,532)		
	MetS n = 765 [57]	Non-MetS n = 604 [43]	Standardized difference	MetS n = 1,038 [68]	Non-MetS n = 494 [38]	Standardized difference
Age ^a^	68.9 (9.1)	68.0 (10.6)	–0.08	67.3 (9.4)	67.0 (9.9)	–0.03
Female sex	431 [56]	313 [52]	0.09	581 [56]	291 [59]	–0.06
Indication						
Primary osteoarthritis	720 [94]	549 [91]	–0.1	849 [82]	366 [74]	–0.2
Other	34 [5]	52 [9]	0.1	186 [18]	125 [25]	0.2
Missing	11 [1]	3 [0]	–0.1	3 [0]	3 [1]	0.05
Charlson index						
Low, 0	550 [72]	453 [75]	0.07	743 [72]	356 [72]	0.02
Medium, 1–2	161 [21]	129 [21]	0.01	233 [22]	113 [23]	0.01
High, ≥3	54 [7]	22 [4]	–0.2	62 [6]	24 [5]	–0.05
Body mass index ^a^	29.1 (4.2)	25.3 (3.7)	–1.0	31.4 (5.5)	26.6 (4.0)	–1.0
Missing	8 [1]	2 [0]	–0.1	2 [0]	2 [0]	0.07
Abdominal circumference, cm ^a^	101.8 (10.9)	90.2 (10.9)	–1.1	106.1 (12.3)	93.3 (12.2)	–1.0
Missing	9 [1]	2 [0]	0.2	15 [1]	13 [3]	0.08
Factors of metabolic syndrome						
Elevated fasting triglycerides	291 [38]	41 [7]	–0.8	351 [34]	22 [5]	–0.8
Decreased high-density lipoprotein	176 [23]	23 [4]	–0.6	222 [21]	15 [3]	–0.6
Hypertension	742 [97]	462 [77]	–0.6	1,017 [98]	387 [78]	–0.6
Elevated fasting P-glucose or diabetes	699 [91]	229 [38]	–1.4	970 [93]	151 [31]	–1.7

a Mean (SD).

**Table 5:** Length of stay (LOS) and differences between groups using multivariate linear regression crude and adjusted for age

LOS, days	MetS n = 1,803	Non-MetS n = 1,098	Difference between groups	
			mean days (CI)	P value
Median (IQR)	1 (1–2)	1 (1–2)		
[range]	[0–28]	[0–9]		
Mean (SD)	1.7 (1.6)	1.5 (1.1)	crude 0.20 (0.10–0.29)	< 0.001
			adjusted 0.20 (0.10–0.29)	< 0.001

**Table 6:** Length of stay and differences between groups stratified by joint, using multivariate linear regression adjusted for age

LOS, days	Hip procedures (n = 1,369)			P value	Knee procedures (n = 1,532)			P value
	MetS n = 765	Non-MetS n = 604	Difference between groups mean days (CI)		MetS n = 1,038	Non-MetS n = 494	Difference between groups mean days (CI)	
Median (IQR)	1 (1–2)	1 (1–2)			1 (1 to 2)	1 (1 to 2)		
[range]	[0–28]	[0–9]			[0 to 24]	[0 to 9]		
Mean (SD)	1.4 (1.4)	1.3 (0.8)	crude 0.10 (–0.01 to 0.21)	0.09	1.9 (1.6)	1.8 (1.3)	crude 0.16 (0.01 to 0.31)	0.04
			adjusted 0.09 (–0.03 to 0.20)	0.13			adjusted 0.16 (0.01 to 0.31)	0.04

The overall readmission rate for medical complications within 30 days was 1.2% and within 90 days 2.3%. 2 patients had 2 separate readmissions within 90 days, 1 patient at 30 days and an additional patient at 90 days. When stratifying by MetS, these patients contributed with 0.8% of the overall 30-day readmissions and 1.7% of the 90-day readmissions, leaving a readmission rate in the non-MetS group of 0.4% at 30 days and 0.6% at 90 days (Tables 7 and 8, see Appendix). Patients with MetS did not have an increased risk of readmission within 30 days (adjusted RR 1.1, CI 0.9–1.4; P = 0.3), but this changed to a 20% increased risk of readmission within 90 days (adjusted RR 1.2, CI 1.0–1.4; P = 0.02) compared with patients without MetS (Table 7). Cardiovascular complications seemed to be the most frequent cause of readmission, reaching 0.5% at 30 days and 1.0% at 90 days (Table 7). The MetS group accounted for 77% of the cardiovascular complications at 30 days and 80% at 90 days.

**Table 7:** Risk of readmission, crude and adjusted for age, number and type of event in readmission at 30 and 90 days after surgery

Hospitalization event	Relative risk crude (CI)	P	Relative risk adjusted (CI)	P	MetS n (%)	Non-MetS n (%)
Readmission at 30 days						
Any event	1.1 (0.9–1.4)	0.3	1.1 (0.9–1.4)	0.3	24 (0.8)	10 (0.3)
Thromboembolism	1.2 (0.7–2.1)	0.5	1.2 (0.7–2.1)	0.6	3 (0.1)	1 (0.03)
Stroke	^a^		^a^		0 (0)	0 (0)
Cardiovascular	1.2 (0.9–1.7)	0.2	1.2 (0.9–1.7)	0.2	10 (0.3)	3 (0.1)
Pulmonary	0.9 (0.5–1.8)	0.8	0.9 (0.5–1.7)	0.8	4 (0.1)	3 (0.1)
Renal	^a^		^a^		1 (0.03)	0 (0)
Urinary tract infection	0.8 (0.2–3.2)	0.8	0.8 (0.2–3.2)	0.8	1 (0.03)	1 (0.03)
Other infections	1.2 (0.8–1.0)	0.4	1.2 (0.8–1.8)	0.4	6 (0.2)	2 (0.07)
Readmission at 90 days						
Any event	1.2 (1.0–1.4)	0.01	1.2 (1.0–1.4)	0.02	50 (1.7)	17 (0.6)
Thromboembolism	0.9 (0.5–1.7)	0.8	0.9 (0.5–1.7)	0.8	4 (0.1)	3 (0.1)
Stroke	^a^		^a^		3 (0.1)	(0)
Cardiovascular	1.3 (1.1–1.6)	0.01	1.3 (1.1–1.5)	0.01	24 (0.8)	6 (0.2)
Pulmonary	0.9 (0.5–1.8)	0.8	0.9 (0.5–1.7)	0.8	4 (0.1)	3 (0.1)
Renal	0.8 (0.2–2.7)	0.7	0.8 (0.2–2.7)	0.7	2 (0.07)	2 (0.07)
Urinary tract infection	1.3 (0.9–1.9)	0.1	1.3 (0.9–1.9)	0.1	5 (0.2)	1 (0.03)
Other infections	1.2 (0.9–1.7)	0.3	1.2 (0.9–1.7)	0.3	9 (0.3)	3 (0.1)

a Events did not occur in both groups.

**Table 8:** Risk of readmission stratified by joint, crude and adjusted for age, number and type of event in readmission at 30 and 90 days after surgery

Hospitalization event	Hip procedures (n=1,369)						Knee procedures (n=1,532)					
	Relative risk crude (CI)	P	Relative risk adjusted (CI)	P	MetS	Non-MetS	Relative risk crude (CI)	P	Relative risk adjusted (CI)	P	MetS n (%)	Non-MetS n (%)
					n (%)	n (%)						
Readmission at 30 days												
Any event	1.0 (0.6–1.5)	0.8	0.9 (0.6–1.5)	0.8	8 (0.6)	7 (0.5)	1.3 (1.0–1.5)	0.03	1.3 (1.0–1.5)	0.03	16 (1.0)	3 (0.2)
Thromboembolism	^a^		^a^		0 (0)	1 (0.07)	^a^		^a^		3 (0.2)	0 (0)
Stroke	^a^		^a^		0 (0)	0 (0)	^a^		^a^		0 (0)	0 (0)
Cardiovascular	1.3 (0.8–2.4)	0.3	1.3 (0.7–2.4)	0.4	3 (0.2)	1 (0.07)	1.2 (0.8–1.6)	0.4	1.1 (0.8–1.6)	0.5	7 (0.5)	2 (0.1)
Pulmonary	0.6 (0.1–2.9)	0.5	0.6 (0.1–2.9)	0.5	1 (0.07)	2 (0.1)	1.1 (0.6–2.0)	0.7	1.1 (0.6–2.0)	0.8	3 (0.2)	1 (0.07)
Renal	^a^		^a^		0 (0)	1 (0.07)	^a^		^a^		1 (0.07)	0 (0)
Urinary tract infection	^a^		^a^		1 (0.07)	0 (0)	^a^		^a^		0 (0)	1 (0.07)
Other infections	1.1 (0.5–2.2)	0.9	1.1 (0.5–2.2)	0.0	3 (0.2)	2 (0.1)	^a^		^a^		3 (0.2)	0 (0)
Readmission at 90 days												
Any event	1.1 (0.8–1.4)	0.7	1.1 (0.8–1.4)	0.9	20 (1.5)	14 (1.0)	1.4 (1.2–1.5)	< 0.001	1.4 (1.2–1.5)	< 0.001	30 (2.0)	3 (0.2)
Thromboembolism	^a^		^a^		0 (0)	3 (0.2)	^a^		^a^		4 (0.3)	0 (0)
Stroke	^a^		^a^		1 (0.07)	0 (0)	^a^		^a^		2 (0.1)	0 (0)
Cardiovascular	1.2 (0.9–1.8)	0.3	1.2 (0.8–1.8)	0.3	9 (0.7)	4 (0.3)	1.3 (1.1–1.6)	0.003	1.3 (1.1–1.6)	0.004	15 (1.0)	2 (0.1)
Pulmonary	0.6 (0.1–3.0)	0.5	0.6 (0.1–3.0)	0.5	1 (0.07)	2 (0.1)	1.1 (0.6–2.0)	0.7	1.1 (0.6–2.0)	0.8	3 (0.2)	1 (0.07)
Renal	0.6 (0.1–3.8)	0.6	0.6 (0.1–3.8)	0.6	1 (0.07)	2 (0.1)	^a^		^a^		1 (0.07)	0 (0)
Urinary tract infection	^a^			^a^	4 (0.3)	0 (0)	0.7 (0.2–2.9)	0.7	0.7 (0.2–2.9)	0.7	1 (0.07)	1 (0.07)
Other infections	1.0 (0.5–2.0)	0.9	1.0 (0.5–2.0)	0.9	4 (0.3)	3 (0.2)	^a^		^a^		5 (0.3)	0 (0)

a Events did not occur in both groups.

## Discussion

We aimed to investigate the length of stay (LOS) and risk of readmission at 30 and 90 days after hip and knee arthroplasty in patients with and without MetS. We found that patients with MetS had longer LOS (0.2 days, which in a clinical setting will have minimal consequences) and a 20% higher readmission risk within 90 days.. We consider the LOS to be the same in both groups and of similar length to that in other fast-track settings [22]. The range in LOS provides important information, suggesting that patients with MetS are likely to have a greater variation in the LOS, making planning in an elective surgery setting more unpredictable. This could reflect the possible difficulties managing clinically unknown comorbidities postoperatively. In relation to this, previous studies on delayed discharge after THA and TKA suggest that comorbidity and BMI > 30 are predictors of special interest [23,24]. This emphasizes that MetS could be a better reflection of the patient’s comorbidity burden, in contrast to obesity as a single factor.

We showed a 90-day readmission rate of 2.3%, which we consider low, although similar results have been found in other fast-track facilities [25,26]. Patients with MetS accounted for more than 75% of the readmissions, adding to the increasing evidence of comorbidity as an important factor for readmission in patients undergoing hip and knee arthroplasty [27]. Patients with MetS have an increased risk of deep vein thrombosis [28], and in relation to surgery MetS has been found to increase the risk of atrial fibrillation, pulmonary oedema, arrhythmias, and cardiac arrest [29]. Thus, our results align with previous research on complications among MetS patients [8,29] and highlight a higher 90-day readmission rate. This should be considered in light of 62% of our study population having MetS, which is in line with existing literature in patients with OA [6]. Previous research on complications in patients with MetS undergoing hip or knee arthroplasty finds a 5–10% prevalence of MetS in the study population [30,31]. This might be highly underreported and could be explained by inconsistent use of MetS definitions and information bias in studies depending on patients’ self-reporting of comorbidities. Previous studies on the transition to fast-track methodology in hip and knee arthroplasty did not find any negative impact on complication rate, but only a reduction in LOS, suggesting our findings could be comparable in the context of programs in hip and knee arthroplasty that do not utilize a fast-track setting [32].

### Methodological considerations

The high completeness of data in our cohort minimizes the risk of selection bias and makes our results generalizable within the context of a similar population. All patients were eligible for readmission at 30 days, whereas 4 patients had died in the time between 30 and 90 days’ follow-up. In the inclusion period, a total of 1,232 patients declined to participate, introducing a potential selection bias. Relying on existing literature, we would assume that these patients had characteristics similar to the study population, but we did not have the data available. We included simultaneously bilateral procedures, as the literature does not provide unambiguous evidence that these procedures are less safe than staged bilateral surgery [33]. Furthermore, the literature does not suggest any difference in readmission rates between hip and knee arthroplasty [34]. Overall, the readmission events investigated had very few observations. Due to the limited size of the cohort, there is a lack of power making direct comparison between groups unreliable in events with no observations in 1 group. Moreover, we did not compare admission events to similar groups not having surgery, suggesting that we could only hypothesize that the surgical procedure would be related to the readmissions identified. Since we identified readmission events from registry data, there is a limitation in the potential misclassification of readmissions and events. This could have been prevented by manually verifying the events in patient records, which we did not have permission to do. Moreover, as registry data did not differentiate between the type of hospital visit (e.g., outpatient clinic or emergency department), our definition of a readmission could contribute to misclassification and maybe explain the low readmission rate. In the MetS group, 58 patients had a diagnosis of diabetes mellitus type 1, compared with 8 patients in the non-MetS group. This highlights the complexity of the MetS definition and the risk of misclassification because the mechanism of elevated fasting glucose is not considered. The occurrence of MetS in individual patients can vary over time and as the presence of MetS was established at inclusion, there could potentially be a different distribution of MetS among the study population at the time of evaluation at 30 and 90 days after surgery. However, we believe that a potential different distribution would be very small and hence without significant impact on the results. Finally, confounding from other unmeasured factors such as lifestyle behavior and physical activity cannot be neglected.

### Conclusion

We showed that MetS patients undergoing hip or knee arthroplasty face marginally longer hospital stays, but exhibit up to a 20% increased risk of postoperative complications needing readmission within the first 90 days.

In perspective, it is crucial to communicate this during outpatient consultations. It is also crucial to minimize the comorbidity burden, which can be aided by lifestyle behavior changes, presurgical metabolic screening, and comprehensive prereferral evaluations by general practitioners.

## Notes

RRS managed the project, analyzed data, and authored the manuscript. ST reviewed the statistics and the manuscript. LER assisted in the establishment of the project and reviewed the manuscript. CLB assisted in sampling and analyzing blood samples and reviewed the manuscript. CV assisted in establishment and managing the project and reviewed the manuscript.

OPEN, Open Patient data Explorative Network, Odense University Hospital, Region of Southern Denmark is acknowledged for assistance in data curation, and research staff at the Department of Orthopadic Surgery, Lillebaelt Hospital, University Hospital of Southern Denmark, for handling the inclusion of patients.

Handling co-editors: Keijo Mäkelä and Robin Christensen

*Acta* thanks Esa Jämsen and Tom Marco van Raaij for help with peer review of this manuscript.
